# Experimentation and Hospitals

**Published:** 1899-03-25

**Authors:** 


### Editor's Letter-Box.

[Our Correspondents are reminded that prolixity is a great bar to publication, and that brevity of style and conciseness of statement greatly facilitate early insertion.]

### EXPERIMENTATION AND HOSPITALS.

We have received a letter from the Hon. Stephen Coleridge in which he brings forward an instance of a hospital giving a sum year by year to its medical school, as controverting a paragraph in our leading article of March 11th, to which he refers in the following words: "The suggestion is made in that article that 'as a rule, if not invariably, the experimentation is absolutely unconnected with the hospital.'" We do not publish the letter, firstly, because it seems clear that in writing it the Hon. Stephen Coleridge has merely made our article an excuse, and that his real object is to use our columns for the purpose of attacking one of our largest and most useful hospitals, a proceeding to which we decline to be made parties; and, secondly, because we do not appreciate his method of making a partial, and what we venture to call a disingenuous, quotation. Surely he must see that, although in the above quotation he gives the precise words we wrote, the meaning of those words is defined and made evident by the rest of the sentence which he does not quote, and is there shown to be different from the meaning he attributes to them.

The sentence runs as follows: "As a rule, if not invariably, the experimentation is absolutely unconnected with the hospital, is conducted in a different building, and is merely one of the means of study pursued by the holder of a hospital appointment, and pursued quite apart from his discharge of the duties which that appointment imposes upon him."

Now, we ask, what right has the Hon. Stephen Coleridge so to quote that sentence as to make it appear that its veracity is disproved by the discovery of a case in which a hospital gives money towards the support of a medical school, when the sentence quoted in its entirety exactly describes the condition of affairs which exists at the very school to which he alludes? It is the perpetual recurrence of this sort of disingenuous quotation which makes argument with anti-vivisectors so disagreeable and thankless a task.

